# *catena*-Poly[bis(dimethylazanium) [[chloridocopper(II)]-di-μ-chlorido-[chloridocopper(II)]-di-μ-azido-κ^4^ *N*:*N*]]

**DOI:** 10.1107/S1600536811050537

**Published:** 2011-11-30

**Authors:** Wei-Yi Zhang, Li Yang, Jie Liu

**Affiliations:** a Department of Obstetrics and Gynecology, The First Affiliated Hospital of Henan University, of Traditional Chinese Medicine, Zhengzhou, 450008, People’s Republic of China; b Henan Medical College for Staff and Workers, Zhengzhou, 451191, People’s Republic of China; c Department of Urology, Henan Provincial People’s Pospital, Zhengzhou, 450003, People’s Republic of China

## Abstract

The crystal structure of the title complex, {(C_2_H_8_N)[CuCl_2_(N_3_)]}_*n*_, exhibits inorganic chains consisting of Cu(II) cations as well azide and chloride anions. The chains, made up from Cu—Cl—Cu—N—Cu linkages, are aligned parallel to the *c* axis. This architecture is further stabilized by a number of N—H⋯Cl hydrogen bonds involving the protonated charge-compensating dimethyl­amine cations and chloride atoms.

## Related literature

For background to polynuclear complexes, see Goher *et al.* (2000 ▶); Liu *et al.* (2008 ▶); Ribas *et al.* (1994 ▶); Saha *et al.* (2005 ▶); Vicente *et al.* (1993 ▶); Wang *et al.* (2008 ▶). For di- or polyalkyl­amines as templates, see: Cheetham *et al.* (1999 ▶); Hagrman *et al.* (1999 ▶). For related copper(II) complexes, see: Mautner *et al.* (1999 ▶).
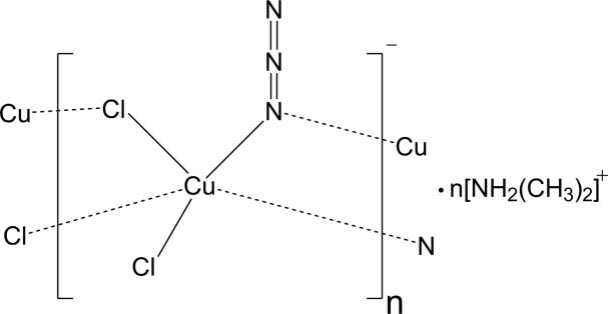


## Experimental

### 

#### Crystal data

(C_2_H_8_N)[CuCl_2_(N_3_)]
*M* *_r_* = 222.57
Monoclinic, 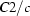

*a* = 15.348 (5) Å
*b* = 11.089 (2) Å
*c* = 10.729 (2) Å
β = 119.73 (2)°
*V* = 1585.7 (7) Å^3^
*Z* = 8
Mo *K*α radiation
μ = 3.35 mm^−1^
*T* = 298 K
0.14 × 0.10 × 0.08 mm

#### Data collection

Bruker APEXII CCD diffractometer
Absorption correction: multi-scan (*SADABS*; Sheldrick, 2003 ▶) *T*_min_ = 0.651, *T*_max_ = 0.775
3510 measured reflections
1811 independent reflections
1251 reflections with *I* > 2σ(*I*)
*R*_int_ = 0.025

#### Refinement

*R*[*F* ^2^ > 2σ(*F* ^2^)] = 0.027
*wR*(*F* ^2^) = 0.066
*S* = 0.94
1811 reflections
85 parameters
13 restraints
H-atom parameters constrained
Δρ_max_ = 0.41 e Å^−3^
Δρ_min_ = −0.38 e Å^−3^

### 

Data collection: *APEX2* (Bruker, 2007 ▶); cell refinement: *SAINT* (Bruker, 2007 ▶); data reduction: *SAINT*; program(s) used to solve structure: *SHELXS97* (Sheldrick, 2008 ▶); program(s) used to refine structure: *SHELXL97* (Sheldrick, 2008 ▶); molecular graphics: *SHELXTL* (Sheldrick, 2008 ▶); software used to prepare material for publication: *publCIF* (Westrip, 2010 ▶).

## Supplementary Material

Crystal structure: contains datablock(s) I, global. DOI: 10.1107/S1600536811050537/zb2020sup1.cif

Structure factors: contains datablock(s) I. DOI: 10.1107/S1600536811050537/zb2020Isup2.hkl

Additional supplementary materials:  crystallographic information; 3D view; checkCIF report

## Figures and Tables

**Table 1:** Selected bond lengths (Å)

Cu1—N1^i^	1.987 (2)
Cu1—N1	2.002 (2)
Cu1—Cl2	2.2527 (8)
Cu1—Cl1	2.2729 (9)
Cu1—Cl1^ii^	2.8860 (13)
Cu1—Cu1^i^	3.1460 (7)

Symmetry codes: (i) 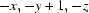; (ii) 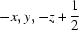.

**Table 2:** Hydrogen-bond geometry (Å, °)

*D*—H⋯*A*	*D*—H	H⋯*A*	*D*⋯*A*	*D*—H⋯*A*
N4—H1⋯Cl1^iii^	0.99	2.41	3.331 (3)	154
N4—H2⋯Cl2^ii^	0.87	2.50	3.257 (3)	146
N4—H2⋯Cl1	0.87	2.82	3.270 (2)	114
N4—H2⋯Cl2	0.87	2.92	3.340 (3)	112

Symmetry codes: (ii) 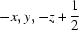; (iii) 
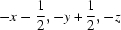
.

**Fig. 1.**
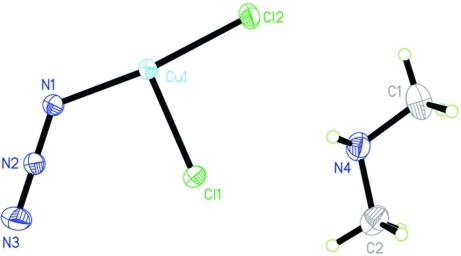
The molecular structure for title compound. Displacement ellipsoids at the 30% probability level. Symmetry codes: (i) -x,-y + 1,-z

## supplementary crystallographic information

### Comment

It is well known that the azide ion is a versatile ligand, and its versatility and efficiency lie in its functionality as a terminal monodentate and a bridging bi-, tri-, and tetradentate ligand. Because of this unique capability, azide attracts a lot of attention in the design of mono- or multidimensional metal-assembled azido complexes. (Vicente *et al.*, 1993; Ribas *et al.*, 1994; Goher *et al.*, 2000; Saha *et al.*, 2005; Liu *et al.*, 2008). Having control over the molecular dimensions and geometry of the metal-ligand moiety in the compounds may lead to the control over their magnetic properties.(Wang *et al.*, 2008). Di- or polyalkylamines, if protonated, could be conveniently used as cationic templates, and they have been widely employed in making metal oxalates, metal phosphates, and oxometalates.(Cheetham *et al.*, 1999; Hagrman *et al.*, 1999).In order to study the coordination behavior of the azide ion and templates, we synthesized herein the title complex [(NH_2_(CH_3_)_2_)(CuN_3_Cl_2_)]_n_. As shown in Figure 1, each asymmetric unit contains one Cu(II) atom, two chloride atoms, one azide atom and one dimethylamine cation. This architecture is further stabilized by a number of N—H···Cl hydrogen bonds involving the protonated charge-compensating dimethylamine cations and chloride atoms.(Figure 2). The bond distances for Cu—N are 1.984 (2) and 2.001 (2) Å, respectively. and the angles for N—Cu—Cl are between 92.86 (6) and 167.48 (6)°. The Cu—Cl bond lengths are 2.2526 (8) Å, 2.2725 (10) Å, respectively. and the bond angles for N—Cu—N and Cl—Cu—Cl are 75.74 (10) and 94.61 (2)°, respectively. These bond distances and bond angles are in agreement with those found in the reported copper compounds(Mautner *et al.*, 1999).

### Experimental

A mixture of methanol and water (1:1, 2 ml) was gently layered on the top of a solution of Cu(ClO_4_)_2_.6H_2_O (37.1 mg, 0.1 mmol) in water (3 ml). A solution of dimethylamine (18 mg, 0.4 mmol), NaN_3_ (13 mg, 0.2 mmol) and hydrochloric acid (40.5 mg, 0.4 mmol, 36%) in methanol (10 ml) was added carefully as the third layer. Green crystals were obtained after 3 weeks, washed with ethanol and ether, and dried in air.

### Refinement

During refinement, H atoms were placed in calculated positions and allowed to ride, with C—H = 0.93 Å and *U*_iso_(H) = 1.2*U*_eq_(C)or *U*_iso_(H) = 1.5*U*_eq_(C).

Data collection: *APEX2*(Bruker, 2007); cell refinemnet: *SAINT* (Bruker, 2007); data reduction: *SAINT*; program(*s*) used to solve structures: *SHELXS97*(Sheldrick, 2008); program(*s*) used to refine structures: *SHELXL97*(Sheldrick, 2008); molecular graphics: *SHELXTL*(Sheldrick, 2008); software used to prepare material for publication: *publCIF*.

### Crystal data

**Table:**

(C_2_H_8_N)[CuCl_2_(N_3_)]	*F*(000) = 888
*M**_r_* = 222.57	*D*_x_ = 1.865 Mg m^−^^3^
Monoclinic, *C*2/*c*	Mo *K*α radiation, λ = 0.71073 Å
*a* = 15.348 (5) Å	Cell parameters from 1037 reflections
*b* = 11.089 (2) Å	θ = 2.6–24.6°
*c* = 10.729 (2) Å	µ = 3.35 mm^−^^1^
β = 119.73 (2)°	*T* = 298 K
*V* = 1585.7 (7) Å^3^	Block, green
*Z* = 8	0.14 × 0.10 × 0.08 mm

### Data collection

**Table:**

Bruker APEXII CCD diffractometer	1811 independent reflections
Radiation source: fine-focus sealed tube	1251 reflections with *I* > 2σ(*I*)
graphite	*R*_int_ = 0.025
phi and ω scans	θ_max_ = 27.5°, θ_min_ = 3.7°
Absorption correction: multi-scan (*SADABS*; Sheldrick, 2003)	*h* = −19→19
*T*_min_ = 0.651, *T*_max_ = 0.775	*k* = −14→14
3510 measured reflections	*l* = −13→13

### Refinement

**Table:**

Refinement on *F*^2^	Secondary atom site location: difference Fourier map
Least-squares matrix: full	Hydrogen site location: inferred from neighbouring sites
*R*[*F*^2^ > 2σ(*F*^2^)] = 0.027	H-atom parameters constrained
*wR*(*F*^2^) = 0.066	*w* = 1/[σ^2^(*F*_o_^2^) + (0.0361*P*)^2^] where *P* = (*F*_o_^2^ + 2*F*_c_^2^)/3
*S* = 0.94	(Δ/σ)_max_ = 0.012
1811 reflections	Δρ_max_ = 0.41 e Å^−^^3^
85 parameters	Δρ_min_ = −0.38 e Å^−^^3^
13 restraints	Extinction correction: *SHELXL*, Fc^*^=kFc[1+0.001xFc^2^λ^3^/sin(2θ)]^-1/4^
Primary atom site location: structure-invariant direct methods	Extinction coefficient: 0.0036 (3)

### Special details

**Table:**

Geometry. All e.s.d.'s (except the e.s.d. in the dihedral angle between two l.s. planes) are estimated using the full covariance matrix. The cell e.s.d.'s are taken into account individually in the estimation of e.s.d.'s in distances, angles and torsion angles; correlations between e.s.d.'s in cell parameters are only used when they are defined by crystal symmetry. An approximate (isotropic) treatment of cell e.s.d.'s is used for estimating e.s.d.'s involving l.s. planes.
Refinement. Refinement of *F*^2^ against ALL reflections. The weighted *R*-factor *wR* and goodness of fit *S* are based on *F*^2^, conventional *R*-factors *R* are based on *F*, with *F* set to zero for negative *F*^2^. The threshold expression of *F*^2^ > σ(*F*^2^) is used only for calculating *R*-factors(gt) *etc*. and is not relevant to the choice of reflections for refinement. *R*-factors based on *F*^2^ are statistically about twice as large as those based on *F*, and *R*- factors based on ALL data will be even larger.

### Fractional atomic coordinates and isotropic or equivalent isotropic displacement parameters (Å^2^)

**Table:**

	*x*	*y*	*z*	*U*_iso_*/*U*_eq_	
Cu1	−0.03408 (2)	0.39407 (3)	0.06367 (3)	0.03133 (14)	
Cl1	−0.14064 (5)	0.39423 (5)	0.15347 (8)	0.03677 (18)	
Cl2	−0.03672 (5)	0.19228 (5)	0.03779 (7)	0.0404 (2)	
N1	−0.04383 (19)	0.57303 (19)	0.0352 (3)	0.0409 (6)	
N2	−0.08322 (18)	0.64469 (19)	0.0760 (3)	0.0388 (6)	
N3	−0.1205 (2)	0.7121 (2)	0.1134 (3)	0.0659 (9)	
N4	−0.1737 (2)	0.1071 (2)	0.1860 (3)	0.0492 (6)	
H1	−0.2164	0.1291	0.0834	0.059*	
H2	−0.1180	0.1479	0.2287	0.059*	
C1	−0.1482 (3)	−0.0211 (3)	0.1877 (4)	0.0631 (9)	
H1A	−0.1133	−0.0496	0.2850	0.095*	
H1B	−0.1062	−0.0303	0.1455	0.095*	
H1C	−0.2087	−0.0669	0.1337	0.095*	
C2	−0.2303 (3)	0.1339 (3)	0.2608 (4)	0.0579 (8)	
H2A	−0.2921	0.0894	0.2167	0.087*	
H2B	−0.2446	0.2186	0.2544	0.087*	
H2C	−0.1911	0.1110	0.3598	0.087*	

### Atomic displacement parameters (Å^2^)

**Table:**

	*U*^11^	*U*^22^	*U*^33^	*U*^12^	*U*^13^	*U*^23^
Cu1	0.0392 (2)	0.02505 (19)	0.0368 (2)	0.00100 (14)	0.02419 (17)	0.00218 (13)
Cl1	0.0405 (4)	0.0355 (4)	0.0434 (4)	0.0016 (3)	0.0277 (3)	0.0042 (3)
Cl2	0.0520 (5)	0.0270 (3)	0.0459 (5)	−0.0020 (3)	0.0271 (4)	−0.0012 (3)
N1	0.0609 (17)	0.0280 (11)	0.0535 (16)	0.0035 (10)	0.0433 (14)	0.0048 (10)
N2	0.0495 (15)	0.0285 (12)	0.0501 (16)	0.0050 (10)	0.0336 (13)	0.0062 (10)
N3	0.087 (2)	0.0483 (17)	0.092 (2)	0.0157 (15)	0.067 (2)	0.0034 (14)
N4	0.0569 (14)	0.0425 (11)	0.0524 (14)	−0.0084 (10)	0.0303 (11)	−0.0003 (9)
C1	0.0676 (17)	0.0421 (14)	0.0641 (17)	−0.0036 (14)	0.0208 (15)	0.0025 (13)
C2	0.0592 (17)	0.0641 (15)	0.0575 (17)	−0.0108 (14)	0.0342 (14)	0.0001 (13)

### Geometric parameters (Å, °)

**Table:**

Cu1—N1^i^	1.987 (2)	N4—C1	1.471 (4)
Cu1—N1	2.002 (2)	N4—C2	1.477 (4)
Cu1—Cl2	2.2527 (8)	N4—H1	0.9931
Cu1—Cl1	2.2729 (9)	N4—H2	0.8693
Cu1—Cl1^ii^	2.8860 (13)	C1—H1A	0.9600
Cu1—Cu1^i^	3.1460 (7)	C1—H1B	0.9600
Cl1—Cu1^ii^	2.8860 (13)	C1—H1C	0.9600
N1—N2	1.205 (3)	C2—H2A	0.9600
N1—Cu1^i^	1.987 (2)	C2—H2B	0.9600
N2—N3	1.129 (3)	C2—H2C	0.9600
			
N1^i^—Cu1—N1	75.87 (10)	N3—N2—N1	179.6 (3)
N1^i^—Cu1—Cl2	95.48 (7)	C1—N4—C2	114.3 (2)
N1—Cu1—Cl2	166.02 (7)	C1—N4—H1	106.0
N1^i^—Cu1—Cl1	167.54 (7)	C2—N4—H1	108.0
N1—Cu1—Cl1	92.79 (7)	C1—N4—H2	108.1
Cl2—Cu1—Cl1	94.60 (3)	C2—N4—H2	107.4
N1^i^—Cu1—Cl1^ii^	94.01 (8)	H1—N4—H2	113.1
N1—Cu1—Cl1^ii^	96.89 (7)	N4—C1—H1A	109.5
Cl2—Cu1—Cl1^ii^	94.63 (2)	N4—C1—H1B	109.5
Cl1—Cu1—Cl1^ii^	92.46 (3)	H1A—C1—H1B	109.5
N1^i^—Cu1—Cu1^i^	38.11 (6)	N4—C1—H1C	109.5
N1—Cu1—Cu1^i^	37.76 (7)	H1A—C1—H1C	109.5
Cl2—Cu1—Cu1^i^	132.67 (3)	H1B—C1—H1C	109.5
Cl1—Cu1—Cu1^i^	130.36 (2)	N4—C2—H2A	109.5
Cl1^ii^—Cu1—Cu1^i^	96.92 (2)	N4—C2—H2B	109.5
Cu1—Cl1—Cu1^ii^	87.54 (3)	H2A—C2—H2B	109.5
N2—N1—Cu1^i^	128.05 (19)	N4—C2—H2C	109.5
N2—N1—Cu1	127.70 (19)	H2A—C2—H2C	109.5
Cu1^i^—N1—Cu1	104.13 (10)	H2B—C2—H2C	109.5

Symmetry codes: (i) −*x*, −*y*+1, −*z*; (ii) −*x*, *y*, −*z*+1/2.

### Hydrogen-bond geometry (Å, °)

**Table:**

*D*—H···*A*	*D*—H	H···*A*	*D*···*A*	*D*—H···*A*
N4—H1···Cl1^iii^	0.99	2.41	3.331 (3)	154.
N4—H2···Cl2^ii^	0.87	2.50	3.257 (3)	146.
N4—H2···Cl1	0.87	2.82	3.270 (2)	114.
N4—H2···Cl2	0.87	2.92	3.340 (3)	112.

Symmetry codes: (iii) −*x*−1/2, −*y*+1/2, −*z*; (ii) −*x*, *y*, −*z*+1/2.
